# Estimation of the number of working population at high-risk of COVID-19 infection in Korea

**DOI:** 10.4178/epih.e2020051

**Published:** 2020-07-09

**Authors:** Juyeon Lee, Myounghee Kim

**Affiliations:** 1 Dalla Lana School of Public Health, University of Toronto, Toronto, ON, Canada; 2 People’s Health Institute, Seoul, Korea

**Keywords:** COVID-19, Infection control, Occupational health, Korea

## Abstract

**OBJECTIVES:**

We aimed to identify occupational groups at high-risk of coronavirus disease 2019 (COVID-19) infection in Korea, to estimate the number of such workers, and to examine the prevalence of protective resources by employment status.

**METHODS:**

Based on the sixth Standard Occupational Classification codes, 2015 census data were linked with data from the fifth Korean Working Conditions Survey, which measured how frequently workers directly come into contact with people other than fellow employees in the workplace.

**RESULTS:**

A total of 30 occupational groups, including 7 occupations from the healthcare and welfare sectors and 23 from other sectors, were classified as high-risk occupational groups involving frequent contact with people other than fellow employees in the workplace (more than half of the working hours). Approximately 1.4 million (women, 79.1%) and 10.7 million workers (46.3%) are employed in high-risk occupations. Occupations with a larger proportion of women are more likely to be at a high-risk of infection and are paid less. For wage-earners in high-risk occupations, protective resources to deal with COVID-19 (e.g., trade unions and health and safety committees) are less prevalent among temporary or daily workers than among those with permanent employment.

**CONCLUSIONS:**

Given the large number of Koreans employed in high-risk occupations and inequalities within the working population, the workplace needs to be the key locus for governmental actions to control COVID-19, and special consideration for vulnerable workers is warranted.

## INTRODUCTION

The coronavirus disease 2019 (COVID-19) pandemic is leading to a socioeconomic crisis in many countries. Korea adopted the test-trace-treat model of containing the spread of COVID-19 at the outset of the pandemic, which allowed the country to avoid a draconian border closure and lockdown including the closing of non-essential workplaces, unlike countries in North America and Europe. However, recent outbreaks of COVID-19 in call centers and warehouses have drawn attention to some flaws and drawbacks of the Korean model of controlling COVID-19. Specifically, the workplace is not currently considered an important locus for public health interventions [1], and the health and safety of workers are not being sufficiently protected. These outbreaks also revealed that precarious employment conditions can be a major obstacle to pandemic control. Although Korea has reduced the economic toll of confinement and lockdown measures [2], it has imposed greater health and safety risks on workers by paying little attention to workplace health and safety in the planning and implementation of pandemic control measures.

Protecting the health and safety of workers is a prerequisite for economic activity to continue without confinement and lockdown measures. However, there is a lack of scientific evidence and policy discussion on the workplaces and workers at high-risk of COVID-19 infection. In 2015, Korea was affected by the outbreak of Middle East Respiratory Syndrome, in which the major mode of transmission was close contact with patients within and between hospitals [3]. At that time, however, the health and safety of healthcare workers received little attention in policy and research. This was due to the lack of recognition that “the hospital is not only a *service space* for patients to be cared and treated, but also a *work space* for healthcare workers to work safely and without risks to their health” [4]. In the recent outbreak of COVID-19 in various workplaces, it was confirmed that in addition to healthcare workers, those employed in other occupational sectors are also vulnerable to contracting COVID-19 and can facilitate the community spread of COVID-19. International researchers have developed lists of occupations at high-risk of COVID-19 infection and estimated the number of workers in these occupations, which were identified based on risk factors such as physical proximity in the workplace, exposure to disease and infections, and contact with others [5-10].

In this study, we aimed to identify occupational groups at high-risk of COVID-19 infection and to estimate the number of workers in these high-risk occupations in Korea. We further estimated the number of workers with a risk of intense exposure among the high-risk occupational groups. The prevalence of protective resources to deal with COVID-19, such as trade unions and health and safety committees in the workplace, was also examined according to employment status.

## MATERIALS AND METHODS

We categorized all of the sixth Standard Occupational Classification (SOC) codes into 2 occupational sectors: the healthcare and welfare sectors and other occupational sectors. Fifty-eight occupations, including 8 occupations from the healthcare and welfare sectors (by 2-digit SOC codes) and 50 occupations from other occupational sectors (by 3-digit SOC codes) were included in the analysis. Originally, “medical and welfare-related service jobs,” including long-term care workers and care aides (code 421 in the sixth SOC and code 42 in the seventh SOC), did not belong to the major group of codes for healthcare and welfare occupations in both the sixth and seventh SOCs (code 24), and such workers have therefore not been counted as healthcare workers for the government’s COVID-19 statistics. Nonetheless, since they are de facto frontline workers who care for patients at a close distance, we categorized them as healthcare and welfare sectors.

Two sources of data were utilized for this analysis: the 20% sample collection of the 2015 census and the fifth Korean Working Conditions Survey (KWCS) (2017). The fifth KWCS data was used to identify occupational groups at high-risk of COVID-19 infection in Korea. The KWCS was designed based on the European Working Conditions Survey with the aim of collecting comparable data on working conditions in Korea. The target sample of 50,000 was extracted using the secondary probability proportion-stratified cluster sample survey to reflect the characteristics of the target population (i.e., all Korean residents aged 15 or older and actively participating in the labor market at the time of the survey, including employees, employers, and self-employed). In order to correctly represent the target population, sample weights were applied for the analysis of the survey data. However, due to the small sample size of the fifth KWCS, the parameter estimation for each of the 58 occupations resulted in considerable uncertainty. Thus, based on the sixth SOC codes, the 20% sample collection of the 2015 census was linked with the fifth KWCS data to estimate the number of workers in high-risk occupational groups. The 20% sample collection of the 2015 census, containing approximately 10 million individuals, is currently the only available data that can be used to estimate the number of workers for each of the SOC codes by detailed occupation code.

Information on the frequency of contact with others (people other than fellow employees) was the only variable available for evaluating the risk of COVID-19 infection for each occupation. Other physical job attributes for evaluating COVID-19 risk, such as physical proximity to fellow employees in the workplace, were not measured in the fifth KWCS. To quantify the frequency of contact with others, we used the following KWCS question: “Does your main paid job involve dealing directly with people who are not fellow employees at your workplace, such as customers, passengers, pupils, patients, etc.?” Respondents could select from the following answers: all of the time; almost all of the time; around three-fourths of the time; around half of the time; around onefourth of the time; almost never; never; don’t know; refuse to reply. Respondents who selected “don’t know” or “refuse to reply” were excluded from the analysis. We scored the responses, with 6 points representing the highest possible risk (all of the time) and 0 points representing the lowest risk (never), and estimated the risk scores (weighted median scores) for each of the 58 SOC codes. Occupations with a risk score equal to or greater than 3 (i.e., more than half of the working hours) were categorized as high-risk.

Meanwhile, the intensity of exposure can vary across high-risk occupations depending on the frequency of contact with others in close proximity. To identify workers at a high-risk of intense exposure, we used the following KWCS question: “Does your main paid job involve lifting or moving people?” Respondents who responded that they did so with a frequency equal to or greater than “around one-fourth of the time” (i.e., more than one-fourth of the working hours) were considered to be at a high-risk of intense exposure to COVID-19 infection. We estimated the prevalence of workers with high-intensity exposure risk in each of the high-risk occupations. Then, the estimated number of workers for each of the high-risk occupations was multiplied by the prevalence to estimate the number of workers with high-intensity exposure risk in each of the high-risk occupations.

Finally, despite the high-risk of infection in some occupations, driven by their physical job attributes, protective resources such as trade unions and health and safety committees in the workplace can mitigate the risk [11,12]. On the contrary, the lack of protective resources provides a mechanism through which the risk of infection can be increased. We attempt to identify more vulnerable workers among wage earners in high-risk occupations by examining the prevalence of protective resources by employment status. The existence of 4 types of protective resources at a company or organization are measured in the fifth KWCS, including (1) a trade union, workers’ council, or a similar committee representing employees; (2) a health and safety representative or committee; (3) a safety management unit or team dealing with safety issues in the organization; and (4) a regular meeting in which employees can express their views about what is happening in the organisation. Respondents could select from the following options: yes, no, don’t know, or refuse to reply. Respondents who selected “don’t know” or “refuse to reply” were excluded from the analysis. We classified employment status into 6 categories, 3 being employers, self-employed, unpaid family workers, and 3 being types of wage earners (permanent, temporary, and daily employment). We calculated the weighted prevalence of each of the 4 protective resources by gender, occupational sector, and employment status (only for wage earners).

## RESULTS

Table 1 shows the median risk scores for each of the 58 occupations. Thirty occupations, including 7 occupations from the healthcare and welfare sectors and 23 from other occupational sectors, were classified as high-risk occupations with frequent contact with others for more than half of the working hours (i.e., median score ≥ 3). All occupations in the healthcare and welfare sector, except for dietitians, had a median score of at least 5, meaning that the core job responsibilities in these occupations involved coming into contact with others for almost all of the working hours. These occupations included medical specialists (physicians), pharmacists, physical therapists, nurses, health and medical-related workers (e.g., emergency medical service [EMS] personnel), social welfare service-related workers, and medical and welfare-related service workers (e.g., long-term care workers and care aides). Other occupational sectors also showed high median risk scores. These included religion-related workers, education professionals, finance and insurance clerks, consulting, statistical and information clerks, hairdressing and wedding service workers, transport and leisure services, cooking and food services, sales, store sales, door-to-door sales, street and telecommunications sales, and transport-related elementary occupations (median ≥ 5). This indicates that high-risk occupations included not only healthcare occupations, which are widely recognized as being at high-risk of COVID-19 infection (e.g., physicians and nurses), but also many often-unrecognized occupations in both the healthcare and other occupational sectors.

**Figure 1.**
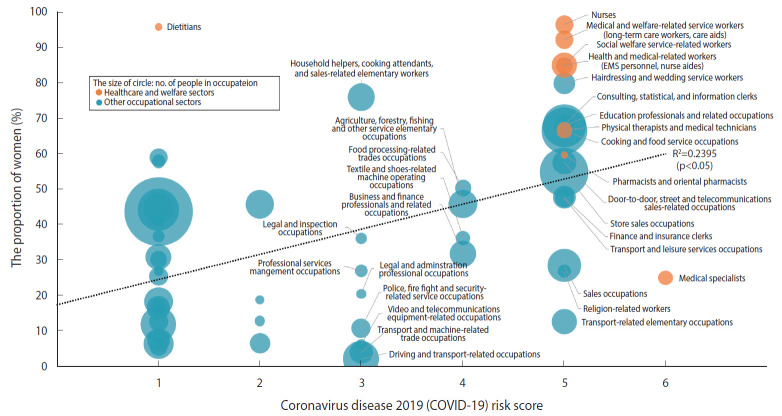
Relationship between the risk score and the proportion of women by occupations. EMS, emergency medical service.

Gender segregation was observed across occupational sectors, as shown in Table 1. For example, in healthcare and welfare sectors, women were under-represented among physicians (25.1%), while the proportions of women were higher than those of men among nurses (96.5%), medical and welfare-related service workers (e.g., long-term care workers and care aides) (92.3%), social welfare service-related workers (85.1%), and health and medical-related workers (e.g., EMS personnel) (84.9%). In other occupational sectors, the proportions of women were much lower than those of men, for example, in driving and transport-related occupations (2.1%), transport and machine-related trade occupations (6.3%), video and telecommunications equipment-related occupations (4.0%), police, firefighter, and security-related service occupations (10.9%), and transport-related elementary occupations (12.7%), while the proportions of women were higher than those of men in, for example, hairdressing and wedding service workers (79.9%), household helpers, cooking attendants, and sales-related elementary workers (76.0%), consulting, statistical, and information clerks (68.1%), and educational professionals and related occupations (67.9%). Figure 1 shows that there was a positive correlation between the proportion of women and the COVID-19 risk score among the 58 occupations (R=0.489; R^2^=0.239; p<0.05). Occupations with a larger share of women were found to be more likely to be at a higher risk of infection.

**Figure 2.**
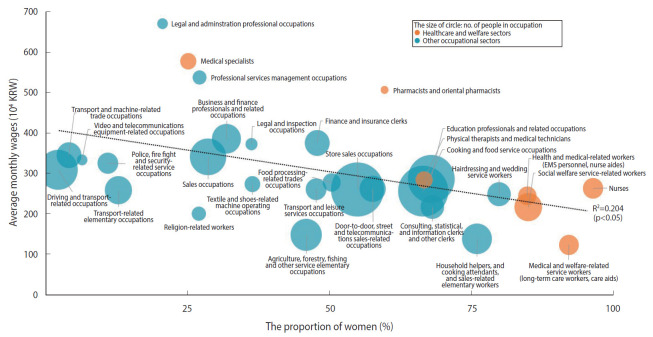
Relationship between the proportion of women and average monthly wages for high-risk occupations. KRW, Korean won; EMS, emergency medical service.

Table 2 shows the gender composition and average monthly wages for each of the 30 high-risk occupations (median ≥ 3). Approximately 1.4 million (women, 79.1%) and 10.7 million workers (46.3%) were employed in high-risk occupations in the healthcare and welfare sectors and in other occupational sectors. Figure 2 shows that there was a negative correlation between the proportion of women in the 30 high-risk occupations and the average monthly wages (R= 0.4523; R^2^= 0.2046; p<0.05). For example, medical and welfare-related service occupations (e.g., long-term care workers and care aides), which were female-dominated occupations (92.3%), had very low average monthly wages (1.24 million Korean won [KRW], equivalent to about 1,000 US dollars), despite their high-risk of infection. Household helpers, cooking attendants, and sales-related elementary occupations also had a large share of women (76%) and low average monthly wages (1.39 million KRW).

Table 3 shows the estimated number of workers with high-intensity exposure risk (i.e., lifting or moving people) in each of the 30 high-risk occupations. Among the 30 high-risk occupations, approximately 540,000 workers (women, 84.7%) in the healthcare and welfare sectors and 1.02 million workers (women, 45.0%) in other occupational sectors had high-intensity exposure risk. In the healthcare and welfare sectors, female-dominated occupations, such as medical and welfare-related service occupations (e.g., long-term care workers and care aides) and nurses had a particularly large share of workers with high-intensity exposure risk (68.6% and 44.2%, respectively). In other occupational sectors, the share of such workers was largest in police, firefighter, and security-related service occupations (25.1%) and household helpers, cooking attendants, and sales-related elementary occupations (19.1%).

Table 4 shows the distribution of employment statuses in the high-risk occupations by gender and occupational sector. Although permanent employment was the most prevalent type in both occupational sectors, the proportion of permanent employment was larger in the healthcare and welfare sectors (77.4%) than in other occupational sectors (50.9%). In both occupational sectors, the proportions of employers and self-employed were larger among men than among women, while the proportions of those carrying out unpaid family work and those with temporary or daily employment were larger among women than among men.

Table 5 shows the prevalence of protective resources among wage earners in the high-risk occupations by gender, occupational sector, and employment status. Men daily workers in the healthcare and welfare sectors were excluded from the analysis due to the small number of these respondents (n=2). The overall prevalence of protective resources was very low for both genders and across occupational sectors and employment statuses. Except for the men wage earners in the healthcare and welfare sectors, all protective resources to deal with occupational hazards were less sources were exceptionally more prevalent among temporary workers than among those with permanent employment. This is because male wage earners in healthcare and welfare sectors are predominantly physicians, which is a highly paid, specialized occupation with a high social status and better access to protective resources, regardless of employment status. Women wage earners in healthcare and welfare sectors had a higher prevalence of all protective resources except trade unions, workers’ councils, or committees representing employees than those in other occupational sectors. In the non-healthcare and welfare sectors, protective resources were less prevalent among women than among men, even with the same employment status.

## DISCUSSION

This study identified occupations in healthcare and welfare and other sectors at high-risk of COVID-19 infection and estimated the number of workers in these high-risk occupations. In addition to 7 occupations in the healthcare and welfare sectors, 23 occupations were identified in other occupational sectors that involve having contact with people other than fellow employees for more than half of the working hours. Furthermore, among the 30 high-risk occupations, the number of workers with high-intensity exposure risk was estimated to be 540,000 in the healthcare and welfare sectors and 1.02 million in other occupational sectors. The results underscore the need for the workplace to be a key locus for governmental actions to control the COVID-19 pandemic and for the government to concentrate its efforts on establishing systems for the management, control, and regulation of occupational health and safety, especially for high-risk occupations. Above all, we argue that the government should collect detailed occupation-related information when tracing the source of infections through epidemiological investigations.

Previous studies from other countries have also reported lists of occupations at high-risk of COVID-19 infection, with similar findings to those of our study. Backer et al. [5] estimated “the number of United States workers, across all occupations, exposed to disease or infection at work more than once a month”. Higher proportions of exposed workers were found not only in the healthcare sector, but also in other sectors, including protective service occupations (e.g., police officers, correctional officers, firefighters), personal care and service occupations, and community and social services occupations. Based on the data from 6 Asian countries, Lan et al. [13] reported that while the high-risk occupations during the early transmission period included shop salespersons, car, taxi, and van drivers, construction laborers, religious professionals, tour guides, and receptionists, those during the late transmission period included health professionals, car, taxi, and van drivers, domestic housekeepers, police officers, and religious professionals.

It should be noted that only 1 physical job attribute (contact with people other than fellow employees) was taken into account in this analysis to identify the occupations with a high-risk of infection. Thus, our list of high-risk occupations is not fully comprehensive, as it is well known that COVID-19 can be easily spread at highly crowded workplaces, as is evident in the recent outbreaks in call centers and warehouses in Korea. This observation is not limited only to Korea. Globally, workplaces have become the center of COVID-19 outbreaks, including call centers in the Philippines [14] and meat processing factories in United States [15], Germany [16], Ireland [17], and Canada [18]. These outbreaks underscore the importance of physical proximity (density), ventilation, and hygiene and sanitary installations in the workplace, as well as contact with others. However, such information was not collected in the fifth KWCS. In order to proactively identify high-risk workplaces and take preventive measures against COVID-19, additional information on working conditions, such as the density, ventilation, and hygiene and sanitary installations is needed. In developing preparedness plans for the next pandemic or emerging infectious diseases, a closer investigation of the working environment is needed.

It should also be pointed out that there are many other occupations which have the potential of being at high-risk of infection. For example, Peccia et al. [19] found severe acute respiratory syndrome coronavirus 2 (SARS-CoV-2) RNA in municipal sewage sludge samples and demonstrated that its concentrations can provide timely information on outbreak dynamics in a community. Such findings raise the possibility that workers at sewage treatment plants may be exposed to a risk of COVID-19 infection. In our additional analysis of the fifth KWCS data, the water treatment and recycling-related operating occupation was the only occupation that involves handling or being in direct contact with materials that can be infectious, such as waste, bodily fluids, and laboratory materials, for more than one-fourth of the working hours. As such, consideration should be given to occupations that may be at risk of infection, even if they do not involve frequent contact with other people.

The characteristics of high-risk occupations in terms of gender, wages, and protective resources need to be better understood and reflected in governmental actions to control COVID-19. Occupations with a larger proportion of women are more likely to be at a higher risk of infection and paid less. The social value of low-wage and high-risk occupations (e.g., long-term care workers and care aides) needs to be reappraised in the post-COVID-19 era, and special consideration for those vulnerable workers is be warranted. Furthermore, this study points out inequalities in protective resources according to employment status. Among wage earners in the high-risk occupations, protective resources were less prevalent among temporary or daily workers than among those with permanent employment. Under the existing Occupational Health and Safety Act (OHS Act), any workplace (with some exceptions) that regularly employs fewer than 100 workers is not required to have a health and safety committee or designate persons to be in general charge of health and safety. Due to these loopholes in the existing OHS Act, workers in small and medium-sized enterprises and with precarious employment remain unprotected. To protect those workers and to prevent the community spread of COVID-19 by those workers, the government needs to ensure access to protective resources for all workers, through which they can effectively deal with safety issues occurring in the workplace.

The COVID-19 pandemic is changing the paradigm of high-risk occupations. Prior to the COVID-19 pandemic, occupations in manufacturing and construction, with higher rates of typical occupational injuries, were deemed as high-risk occupations. The Supplementary Material 1 presents the proportions of workers, across all 58 occupations, who thought that their health and safety were at risk because of their work. Notable occupations with higher proportions of workers who considered themselves to be “at risk” included metal forming-related technical occupations, construction and mining-related elementary occupations, and skilled fishery occupations. Most of the occupations at high-risk of infection identified in this study based on the frequency of contact with others have a low proportion of workers, less than 10%, who think their health and safety are at risk because of their work. The end of COVID-19 does not mean that high-risk occupations will become low-risk occupations. Rather, COVID-19 has raised the need for social protection for workers who are employed in occupations with physical job attributes such as frequent contact with others and physical proximity in the workplace that can potentially put their health and safety at risk.

## Figures and Tables

**Table 1.** Coronavirus disease 2019 (COVID-19) risk scores and the estimated number of workers by occupations

Sixth SOC codes		Risk score^1^	Total, n^2^	Women, n (%)^2^
Healthcare and welfare sectors (by 3-digit codes)				
	Medical specialists	6	145,878	36,574 (25.1)
	Pharmacists and oriental pharmacists	5	35,541	21,232 (59.7)
	Nurses	5	227,168	219,301 (96.5)
	Physical therapists and medical technician	5	158,096	105,461 (66.7)
	Health and medical-related workers (EMS personnel, nurse aides)	5	186,996	158,775 (84.9)
	Social welfare service-related workers	5	430,185	366,009 (85.1)
	Medical and welfare-related service workers (long-term care workers, care aides)	5	222,830	205,581 (92.3)
	Dietitians	1	37,812	36,228 (95.8)
Other occupational sectors (by 2-digit codes)				
	Religion-related workers	5	111,556	30,016 (26.9)
	Education professionals and related occupations	5	1,235,726	839,663 (67.9)
	Finance and insurance clerks	5	354,937	169,706 (47.8)
	Consulting, statistical and information clerks and other clerks	5	313,483	213,613 (68.1)
	Hairdressing and wedding service workers	5	308,603	246,724 (79.9)
	Transport and leisure services occupations	5	249,609	118,839 (47.6)
	Cooking and food service occupations	5	1,415,853	942,284 (66.6)
	Sales occupations	5	737,803	210,655 (28.6)
	Store sales occupations	5	1,576,184	865,985 (54.9)
	Door to door, street and telecommunications sales-related occupations	5	384,429	221,404 (57.6)
	Transport-related elementary occupations	5	426,099	54,186 (12.7)
	Business and finance professionals and related occupations	4	473,382	150,553 (31.8)
	Food processing-related trades occupations	4	178,102	89,709 (50.4)
	Textile and shoes-related machine operating occupations	4	138,160	50,227 (36.4)
	Agriculture, forestry, fishing and other service elementary occupations	4	544,583	249,937 (45.9)
	Professional services management occupations	3	109,368	29,549 (27.0)
	Legal and administration professional occupations	3	64,662	13,248 (20.5)
	Legal and inspection occupations	3	81,923	29,676 (36.2)
	Police, fire fight and security-related service occupations	3	245,764	26,676 (10.9)
	Transport and machine-related trade occupations	3	358,365	14,284 (4.0)
	Video and telecommunications equipment-related occupations	3	65,052	4,105 (6.3)
	Driving and transport-related occupations	3	868,592	17,887 (2.1)
	Household helpers, cooking attendants, and sales-related elementary workers	3	497,271	377,984 (76.0)
	Sales and customer service managers	2	54,666	10,288 (18.8)
	Culture, arts and sports professionals and related occupations	2	547,027	250,232 (45.7)
	Wood and furniture, musical instrument and signboard-related trade occupations	2	73,208	9,383 (12.8)
	Electric and electronic-related trade occupations	2	279,374	18,455 (6.6)
	Administrative and business support management occupations	1	74,630	12,263 (16.4)
	Construction, electricity and production-related managers	1	45,269	2,784 (6.1)
	Science professionals and related occupations	1	99,892	36,726 (36.8)
	Information and communication professionals and technical occupations	1	367,406	60,871 (16.6)
	Engineering professionals and technical occupations	1	846,303	100,155 (11.8)
	Administration and accounting-related occupations	1	3,171,132	1,387,086 (43.7)
	Agricultural, livestock-related skilled occupations	1	1,155,422	511,413 (44.3)
	Skilled fishery occupations	1	58,959	15,810 (26.8)
	Textile, clothing and leather relates trade occupations	1	221,280	130,616 (59.0)
	Metal forming-related trade occupations	1	218,049	12,228 (5.6)
	Construction and mining-related trade occupations	1	595,404	37,916 (6.4)
	Other technical occupations	1	148,804	25,515 (17.1)
	Food processing-related operating occupations	1	121,563	50,295 (41.4)
	Chemical-related machine operating occupations	1	239,152	61,025 (25.5)
	Metal and non-metal-related operator occupations	1	254,918	32,329 (12.7)
	Machine production and related machine operators	1	542,978	99,427 (18.3)
	Electrical and electronic-related machine occupations	1	440,371	136,174 (30.9)
	Water treatment and recycling-related operating occupation	1	37,583	3,572 (9.5)
	Wood, printing and other machine operating occupations	1	197,719	59,849 (30.3)
	Construction and mining-related elementary occupations	1	339,473	24,671 (7.3)
	Production-related elementary occupations	1	123,769	71,679 (57.9)
	Clean and guard-related elementary occupations	1	615,971	275,305 (44.7)
	Skilled forestry occupations	0	5,351	717 (13.4)

SOC, Standard Occupational Classification; EMS, emergency medical service. Data from: ^1^The fifth Korean Working Conditions Survey (2017) and the weighted median score. ^2^The 20% sample of the 2015 census.

**Table 2.** The estimated number of workers and average monthly income by occupations among high-risk groups

High-risk occupations		Total, n	Women, n (%)^1^	Average monthly wages (10^4^ KRW)^2^
Healthcare and welfare sectors (by 3-digit codes)				
	Medical specialists	145,878	36,574 (25.1)	581
	Pharmacists and oriental pharmacists	35,541	21,232 (59.7)	509
	Nurses	227,168	219,301 (96.5)	265
	Physical therapists and medical technicians	158,096	105,461 (66.7)	286
	Health and medical-related workers (EMS personnel, nurse aides)	186,996	158,775 (84.9)	246
	Social welfare service-related workers	430,185	366,009 (85.1)	218
	Medical and welfare-related service workers (long-term care workers, care aides)	222,830	205,581 (92.3)	124
	Total no. of employed in high-risk occupations	1,406,694	1,112,933 (79.1)	
Other occupational sectors (by 2-digit codes)				
	Religion-related workers	111,556	30,016 (26.9)	202
	Education professionals and related occupations	1,235,726	839,663 (67.9)	288
	Finance and insurance clerks	354,937	169,706 (47.8)	378
	Consulting, statistical and information clerks and other clerks	313,483	213,613 (68.1)	219
	Hairdressing and wedding service workers	308,603	246,724 (79.9)	250
	Transport and leisure services occupations	249,609	118,839 (47.6)	263
	Cooking and food service occupations	1,415,853	942,284 (66.6)	258
	Sales occupations	737,803	210,655 (28.6)	343
	Store sales occupations	1,576,184	865,985 (54.9)	262
	Door-to-door, street and telecommunications sales-related occupations	384,429	221,404 (57.6)	264
	Transport-related elementary occupations	426,099	54,186 (12.7)	260
	Business and finance professionals and related occupations	473,382	150,553 (31.8)	388
	Food processing-related trades occupations	178,102	89,709 (50.4)	279
	Textile and shoes-related machine operating occupations	138,160	50,227 (36.4)	276
	Agriculture, forestry, fishing and other service elementary occupations	544,583	249,937 (45.9)	149
	Professional services management occupations	109,368	29,549 (27.0)	540
	Legal and administration professional occupations	64,662	13,248 (20.5)	674
	Legal and inspection occupations	81,923	29,676 (36.2)	374
	Police, fire fight and security-related service occupations	245,764	26,676 (10.9)	327
	Transport and machine-related trade occupations	358,365	14,284 (4.0)	347
	Video and telecommunications equipment-related occupations	65,052	4,105 (6.3)	336
	Driving and transport-related occupations	868,592	17,887 (2.1)	311
	Household helpers, cooking attendants, and sales-related elementary workers	497,271	377,984 (76.0)	139
	Total no. of employed in high-risk occupations	10,739,506	4,966,910 (46.3)	-

KRW, Korean won; EMS, emergency medical service. Data from: ^1^The 20% sample of the 2015 census. ^2^The fifth Korean Working Conditions Survey (2017); Sample weights were applied.

**Table 3.** The estimated number of workers with high-intensity exposure risk by occupations among high-risk groups

High-risk occupations		High-intensity exposure risk		
		Total, %^1^	Total, n^2^	Women, n^3^
Healthcare and welfare sectors (by 3-digit codes)				
	Medical specialists	17.7	25,844	6,480
	Pharmacists and oriental pharmacists	1.7	593	354
	Nurses	44.2	100,463	96,984
	Physical therapists and medical technicians	31.3	49,437	32,978
	Health and medical-related workers (EMS personnel, nurse aides)	32.7	61,200	51,964
	Social welfare service-related workers	36.6	157,324	133,854
	Medical and welfare-related service workers (long-term care workers, care aides)	68.6	152,944	141,105
	Total no. of workers exposed to high-intensity risk		547,806	463,719
Other occupational sectors (by 2-digit codes)				
	Religion-related workers	2.9	3,258	877
	Education professionals and related occupations	9.2	113,390	77,047
	Finance and insurance clerks	5.3	18,847	9,012
	Consulting, statistical and information clerks and other clerks	5.8	18,152	12,369
	Hairdressing and wedding service workers	12.4	38,222	30,558
	Transport and leisure services occupations	8.0	19,854	9,452
	Cooking and food service occupations	8.9	125,369	83,436
	Sales occupations	4.5	33,154	9,466
	Store sales occupations	8.6	135,031	74,188
	Door to door, street and telecommunications sales-related occupations	7.3	27,901	16,069
	Transport-related elementary occupations	12.6	53,556	6,811
	Business and finance professionals and related occupations	5.0	23,633	7,516
	Food processing-related trades occupations	12.1	21,585	10,872
	Textile and shoes-related machine operating occupations	12.9	17,873	6,497
	Agriculture, forestry, fishing and other service elementary occupations	7.3	39,591	18,170
	Professional services management occupations	8.2	8,969	2,423
	Legal and administration professional occupations	2.9	1,848	379
	Legal and inspection occupations	4.3	3,485	1,262
	Police, fire fight and security-related service occupations	25.1	61,740	6,701
	Transport and machine-related trade occupations	13.2	47,471	1,892
	Video and telecommunications equipment-related occupations	9.9	6,423	405
	Driving and transport-related occupations	12.3	106,435	2,192
	Household helpers, cooking attendants, and sales-related elementary workers	19.1	94,915	72,147
	Total no. of workers exposed to high-intensity risk		1,020,704	459,744

EMS, emergency medical service. Data from: ^1^The fifth Korean Working Conditions Survey (2017); The weighted prevalence of workers with high-intensity exposure risk. ^2^The estimated number of workers for each of the 30 high-risk occupations (see Table 2) was multiplied by the weighted prevalence; The 20% sample of the 2015 census. ^3^The number of workers with high-intensity exposure risk was multiplied by the percentage of women for the 30 high-risk occupations (see Table 2); The 20% sample of the 2015 census.

**Table 4.** Employment status of respondents in high-risk occupations by gender

Employment status		Total	Men	Women
Healthcare and welfare sectors				
	Employers	106,472 (6.1)	71,984 (21.4)	34,488 (2.4)
	Self-employed	41,606 (2.4)	28,353 (8.4)	13,253 (0.9)
	Unpaid family workers	4,104 (0.2)	0 (0.0)	4,104 (0.3)
	Permanent workers	1,356,084 (77.4)	216,140 (64.2)	1,139,944 (80.5)
	Temporary workers	202,193 (11.5)	19,390 (5.8)	182,803 (12.9)
	Daily workers	42,401 (2.4)	621 (0.2)	41,780 (2.9)
	Total	1,752,860 (100)	336,488 (100)	1,416,372 (100)
Other occupational sectors				
	Employers	1,033,972 (8.2)	704,234 (10.8)	329,739 (5.4)
	Self-employed	2,651,095 (21.0)	1,534,335 (23.5)	1,116,760 (18.4)
	Unpaid family workers	506,650 (4.0)	57,424 (0.9)	449,226 (7.4)
	Permanent workers	6,406,690 (50.9)	3,536,719 (54.2)	2,869,970 (47.2)
	Temporary workers	1,603,717 (12.7)	540,751 (8.3)	1,062,966 (17.5)
	Daily workers	395,896 (3.1)	146,284 (2.2)	249,612 (4.1)
	Total	12,598,019 (100)	6,519,747 (100)	6,078,272 (100)

Values are presented as number (%). Data from: The fifth Korean Working Conditions Survey (2017); Sample weights were applied.

**Table 5.** Prevalence of protective resources by employment status among wage earners in high-risk occupations

Sectors		Employment statuses	Prevalence of protective resources, %			
			Trade union, workers’ council, or a similar committee representing employees	Health and safety representative or committee	Safety management or team dealing with safety issues in the organization	A regular meeting in which employees can express their views about what is happening in the organisation
Healthcare and welfare sectors						
	Total	Permanent	11.6	18.0	22.3	27.9
		Temporary	4.7	9.8	15.1	19.0
		Daily	1.5	3.3	3.4	2.5
	Men	Permanent	12.5	18.5	24.5	31.4
		Temporary	15.2	28.5	36.8	32.8
		Daily	-	-	-	-
	Women	Permanent	11.5	17.9	21.9	27.3
		Temporary	3.5	7.8	12.7	17.5
		Daily	1.6	3.3	3.4	2.5
Other occupational sectors						
	Total	Permanent	16.7	14.7	21.1	27.6
		Temporary	4.3	5.2	7.4	7.8
		Daily	2.0	3.7	5.2	5.7
	Men	Permanent	20.6	18.1	25.4	31.2
		Temporary	6.9	6.7	9.2	8.1
		Daily	3.7	6.4	9.1	9.4
	Women	Permanent	12.0	10.6	15.7	23.3
		Temporary	3.0	4.5	6.5	7.7
		Daily	1.0	2.2	3.0	3.6

Data from: The fifth Korean Working Conditions Survey (2017); Sample weights were applied.

## SUPPLEMENTARY MATERIALS

Korean version is available at http://www.e-epih.org/.
