# Supplementary material for: Estimation of the number of working population at high-risk of COVID-19 infection in Korea
Source: Epidemiol Health. 2020 Jul 9;42:e2020051. doi: 10.4178/epih.e2020051 (PMC7871163; doi:10.4178/epih.e2020051)
Supplement: Supplementary file 1 [file epih-42-e2020051-suppl1.pdf]

Volume: 42, Article ID: e2020051
https://doi.org/10.4178/epih.e2020051

Supplementary Material 1. Prevalence of workers who think their health and safety are at risk because of their work by occupations (%)

Sixth SOC codes	%
Metal Coremakers Related Trade Occupations	49.77
Construction and Mining Related Elementary Occupations	49.74
Skilled Fishery Occupations	45.34
Construction and Mining Related Trade Occupations	37.21
Metal and Nonmetal Related Operator Occupations	35.81
Driving and Transport Related Occupations	35.79
Transport Related Elementary Occupations	33.69
Skilled Forestry Occupations	32.45
Police, Fire Fight and Security Related Service Occupations	31.33
Water Treatment and Recycling Related Operating Occupation	30.79
Other Technical Occupations	29.01
Electric and Electronic Related Trade Occupations	26.40
Transport and Machine Related Trade Occupations	23.55
Wood and Furniture, Musical Instrument and Signboard Related Trade Occupations	22.42
Machine Production and Related Machine Operators	21.90
Video and Telecommunications Equipment Related Occupations	21.12
Chemical Related Machine Operating Occupations	19.80
Wood, Printing and Other Machine Operating Occupations	18.66
Electrical and Electronic Related Machine Occupations	15.31
Science Professionals and Related Occupations	14.71
Textile and Shoes Related Machine Operating Occupations	13.66
Construction, Electricity and Production Related Managers	13.10
Agricultural, Livestock Related Skilled Occupations	13.03
Engineering Professionals and Technical Occupations	11.99
Textile, Clothing and Leather Relates Trade occupations	11.55
Food Processing Related Trades Occupations	11.50
Food Processing Related Operating Occupations	11.19
Physical Therapists and Medical Technologists	11.12
Clean and Guard Related Elementary Occupations	10.77
Administrative and Business Support Management Occupations	10.10
Cooking and Food Service Occupations	9.45
Medical and Welfare Related Service Workers	9.45
Nurses	8.70
Household Chores and Cooking Attendants and Sales Related Elementary Workers	8.26
Medical Specialists	8.09
Agriculture, Forestry, Fishing and Other Service Elementary Occupations	7.82
Hairdressing and Wedding Service Workers	7.51
Transport and Leisure Services Occupations	7.41
Legal and Administration Professional Occupations	7.22
Health and Medical Related Workers	6.91
Production Related Elementary Occupations	5.31
Culture, Arts and Sports Professionals and Related Occupations	4.72
Store Sales Occupations	4.48
Sales Occupations	4.16
Information and Communication Professionals and Technical Occupations	3.99
Religion Related Workers	3.68
Social Welfare Service Related Workers	2.98
Consulting, Statistical and Information Clerks and Other Clerks	2.86
Administration and Accounting Related Occupations	2.60

Dietitians	2.58
Door to Door, Street and Telecommunications Sales Related Occupations	2.52
Business and Finance Professionals and Related Occupations	2.48
Pharmacists and Oriental Pharmacists	2.35
Finance and Insurance Clerks	2.25
Education Professionals and Related Occupations	2.00
Professional Services Management Occupations	1.85
Legal and Inspection Occupations	1.60
Sales and Customer Service Managers	-

Source: The fifth Korean Working Conditions Survey (2017). Sample weights were applied. Occupations in healthcare and welfare sectors by 3-digit codes and in other occupational sectors by 2-digit codes. SOC, Standard Occupational Classification.
