# Supplementary material for: Estimation of the number of working population at high-risk of COVID-19 infection in Korea
Source: Epidemiol Health. 2020 Jul 9;42:e2020051. doi: 10.4178/epih.e2020051 (PMC7871163; doi:10.4178/epih.e2020051)
Supplement: Supplementary file 2 [file epih-42-e2020051-suppl2.docx]

**코로나19 팬데믹, 감염 고위험 직업군 종사자 규모 추정과 특성에 대한 연구**

**Estimation of the number of working population at high risk of COVID-19 infection in South Korea**

Juyeon Lee^1^, Myounghee Kim^2^

^1^Dalla Lana School of Public Health, University of Toronto, Toronto, ON, Canada; ^2^People’s Health Institute, Seoul, Korea

**초록**

Objectives: 본 연구는 다음 세 가지를 목표로 한다. 첫째, 한국에서 코로나19를 비롯한 감염병에 취약한 고위험 직업군을 파악하여 고위험 직업군 종사자의 규모를 추정한다. 둘째, 고위험 직업군 종사자의 성별 구성, 임금 수준, 고용형태를 분석한다. 셋째, 고위험 직업군 종사자의 고용형태에 따른 보호자원 보유율을 비교한다.

Methods: 본 연구는 제 5차 근로환경조사(2017) 원시 자료를 이용하여 감염 고위험 직업군을 파악하고, 이를 인구주택총조사 20% 표본(2015)과 연계하여 고위험 직업군의 종사자 규모를 추정하였다. 한국표준직업분류(6차 개정)를 기반으로 환자와 직접 대면하는 보건의료복지와 그 외 부문으로 구분하여 보건의료복지 8개 직업군과 그 외 업종 50개 직업군, 총 58개 직업군이 분석에 포함되었다. 감염위험을 측정하는 지표로 ‘환자 및 대중 접촉’ 정도를 유추할 수 있는 변수를 활용하였다. 또한, ‘밀접 접촉’ 변수를 이용하여 고위험 직업군 가운데 ‘고강도 위험’에 노출될 가능성이 있는 종사자 규모를 추정하였다.

Results: 감염위험 중위수가 3점 이상인 고위험 직업군은 보건의료복지 7개 직업군, 그 외 업종 23개 직업군이다. 감염위험 중위수가 3점 이상인 고위험 직업군 종사자는 보건의료복지 업종에서 총 140만 명 (여성 79.1%), 그 외 업종에서 총 1,073만 명 (여성 46.3%)으로 추정되었다. 여성 비율이 높은 직업군 대부분이 고위험 직업군에 속하였고 고강도 위험에 노출되는 비율도 높았으며, 월 평균 임금이 낮게 나타났다. 고용 안정성이 높은 상용노동자가 건강과 안전을 보호받을 수 있는 자원이 있다고 응답한 비율이 다른 고용형태에 비해 높게 나타났다.

Conclusions: 고위험 직업군의 사업장에 대해 정부가 안전보건 관리·감독·규제 및 지원 체계를 수립하고, 방역 대책의 중심에 노동자의 안전과 건강 보호를 두는 관점의 전환이 필요하다. 특히 사회적 보호의 사각지대에 있는 임시직, 일용직 등 불안정 고용 노동자와 전형적 ‘여성 일자리’ 종사자에 대한 보호가 절실하다.

**중심 단어**

COVID-19; infection control; occupational health; South Korea

**서론**

코로나19팬데믹이 사회경제적 위기로 이어지고 있다. 한국은 북미나 유럽 국가들과 달리 초기부터 적극적으로 검사와 접촉자 추적을 실시한 결과 국경을 전면적으로 봉쇄하거나 비필수서비스 사업장을 강제폐쇄하는 극단적인 조치를 피할 수 있었다. 하지만 최근 콜센터, 물류센터 등에서 일어난 집단 감염은 한국 방역 조치의 허점을 드러내고 있다. 현재 방역의 중요한 관리 대상에서 사업장이 제외되어 있고[1], 노동자들이 일터에서 안전과 건강을 보호받지 못하고 있다는 점이다. 또한 불안정고용 구조가 방역대응에 걸림돌이 된다는 점도 드러났다. 한국은 경제봉쇄를 피하면서 노동자들이 겪는 경제적 고통은 경감시킨 반면[2], 방역조치의 기획과 실행 과정에 노동안전보건 관점을 충분히 고려하지 않음으로써 노동자들의 안전과 건강 피해를 키우고 있다.

경제가 멈춤없이 지속되기 위한 전제조건은 일터에서 노동자들이 안전과 건강을 충분히 보호받는 것이다. 하지만 한국에서 감염병유행으로 인한 공중보건위기 상황에서 고위험 사업장/노동자에 대한 논의와 학술적 근거, 정책 모두가 부족하다. 2015년 한국에서 발생한 중동호흡기증후군은 감염자와의 밀접접촉에 의한 병원 감염이 주된 경로였지만[3], 당시에도 보건의료 노동자의 직업안전보건 문제는 크게 주목받지 못했다. 방역대책에서 “병원이 환자를 돌보고 보호하는 서비스 공간일 뿐 아니라, 보건의료 노동자들이 안전하고 건강하게 일할 수 있는 노동의 공간이라는 점”이 충분히 인식되지 않았기 때문이다[4]. 이번 코로나19 유행에서도 보건의료 종사자뿐 아니라 여러 직종의 노동자들이 감염병에 취약하고 지역사회전파를 매개할 수 있다는 사실이 확인되었다. 해외 언론과 학계에서는 환자 접촉, 대중 접촉, 사업장 내 밀집도 등 여러 위험요인에 기반해 감염 고위험직업을 목록화하고 노동자 규모를 추정하고 있다[5-10].

본 연구는 다음 세 가지를 목표로 한다. 첫째, 한국에서 코로나19를 비롯한 감염병에 취약한 고위험 직업군을 파악하여 고위험 직업군 종사자의 규모를 추정한다. 둘째, 고위험 직업군 종사자의 고용형태를 분석한다. 셋째, 고위험 직업군 종사자의 고용형태에 따른 보호자원 보유상황을 비교한다. 본 연구는 고위험직업을 목록화하고 노동자 규모를 추정한 선행연구에서 나아가, 노동자가 감염위험으로부터 스스로를 보호할 수 있는 보호자원에 대한 접근성이 직업과 고용형태에 따라 다른지 확인한다.

**연구방법**

제 5차 근로환경조사(Korean Working Conditions Survey, 이하 KWCS)(2017)^[[1]](#endnote-1)^ 원시자료를 이용하여 감염 고위험 직업군을 파악하고, 이를 인구주택총조사 20% 표본(2015)과 연계하여 고위험 직업군의 종사자 규모를 추정하였다. KWCS는 전국의 만15세 이상 근로인구 50,000명을 추출하여 국내 모든 가구 내 만15세 이상 취업자(근로자, 사업주와 자영업자 포함)의 특성을 반영하도록 했으며, 분석에는 가중치를 적용했다. 하지만 근로환경조사는 표본수가 적어 직업군별 모수를 추정하는 데 상당한 불확실성이 있다. 따라서, 고위험 직업군에 속한 실제 인구규모를 보다 정확히 추정하기 위해서 2015 인구주택총조사 20% 표본조사를 활용하였다.^[[2]](#endnote-2)^ 인구주택총조사는 5년을 주기로 시행되며, 심층적인 특성은 전국 가구의 20% 표본에 대해 현장조사로 파악한다. 현재 가용한 최신 자료는 2015년 조사자료이다. 또한, 현재 한국에서 표준직업분류 2, 3자리까지 구분하여 근로인구 규모를 파악할 수 있는 자료는 인구주택총조사 표본조사가 유일하다.

KWCS(2017)에서 직업은 한국표준직업분류(6차 개정)를 기반으로 4자리 세세분류까지 조사한다. 우리는 직업을 크게 환자와 직접 대면하는 보건의료복지와 그 외 부문으로 구분하고, 보건의료복지 8개 직업군과 그 외 부문 50개 직업군, 총 58개 직업군을 분석대상으로 삼았다. 보건의료복지 부문은 3자리 직업분류로, 그 외 부문은 2자리 직업분류로 구분하여 분석하였다. 보건의료복지 부문은 ‘보건사회복지 및 종교관련직 (24)’에서 종교관련직을 제외한 모든 직업군(241-247), 그리고 ‘이미용예식 및 의료보조 서비스직(42)’ 중에서 의료복지 관련 서비스 종사자(421)를 포함한다. 의료복지 관련 서비스 종사자(421)는 간병인과 요양보호사 등 기타 의료복지 관련 서비스 종사원을 포함하며, 이들 직종은 현재 정부의 보건의료인 감염 통계 집계에 포함되지 않지만 환자와의 접촉 강도가 높은 최일선 인력이라는 점에서 보건의료복지 부문으로 분류하였다.

감염병 노출에 위험한 직업군을 확인하기 위해서 환자접촉, 불특정 다수의 대중접촉, 사업장 내 밀집도 등을 고려할 수 있다. 그러나 KWCS에는 사업장 내 직장동료와의 물리적 근접성을 확인할 수 있는 변수가 없다. 따라서 감염위험을 측정하는 지표로 ‘환자 및 대중 접촉’ 정도를 유추할 수 있는 변수를 활용하였다 (문항 Q26-G: 귀하가 하는 일에는 고객, 승객, 학생, 환자와 같은 직장동료가 아닌 사람들을 직접 상대하는 업무가 포함되어 있습니까?). 응답자는 근무시간 중 노출정도에 따라 7개의 범주로 응답하였다 (①근무시간 내내 ②거의 모든 근무시간 ③근무시간 3/4 ④근무시간 절반 ⑤근무시간 1/4 ⑥거의 노출 안 됨 ⑦ 절대 노출 안 됨 ⑧모름/무응답 ⑨ 거절). ‘환자 및 대중 접촉’ 정도가 가장 낮은 범주(⑦)에 0점, 가장 높은 범주(①)에 6점 순으로 부여하였고, 연속형 변수로 분석에 활용하였다. ⑧모름/무응답 혹은 ⑨거절은 분석에서 제외하였다. 감염위험의 중위값이 3점 이상인 직업군, 즉 근무시간 절반 이상 환자 및 대중을 직접 상대하는 직업군을 ‘고위험 직업군’으로 정의하였다.

한편, 환자 및 대중을 직접 상대하는 정도가 높은 고위험 직업군에 속하더라도 감염 노출 강도는 ‘밀접 접촉’ 정도에 따라 다르게 나타날 수 있다. KWCS에서 ‘밀접 접촉’을 유추할 수 있는 변수는 ‘사람을 들어 올리거나 이동시킴’의 정도를 확인하는 Q26-B 문항이 유일했다 (Q26-B: 귀하가 하는 일에는 사람을 들어 올리거나 이동시키는 업무가 포함되어 있습니까?). 이 문항을 이용하여 고위험 직업군 가운데 ‘고강도 위험’에 노출될 가능성이 있는 실제 인구 규모를 추정하였다.

마지막으로, 감염위험에 노출되더라도 예방과 관리에 활용할 수 있는 보호자원이 있다면 감염위험을 피하거나 최소화시킬 수 있다[11-12]. 감염병 발생 시 보호자원의 부재는 감염 위험을 높이는 기전으로 작동할 수 있다. 본 연구는 고위험 직업군에게 마땅히 보장되어야 하는 보호자원의 고용형태별 분포를 확인하여, 고위험 직업군 가운데서도 특히 감염에 취약한 집단을 파악하였다. KWCS (2017)는 노동자의 의견을 대표하거나 노동자의 권익을 위해 활동하는 다음 네 가지 보호자원의 보유 여부에 대해 조사한다. 첫째, 노동조합, 노동자 협의회나 직원을 대표하는 유사위원회(Q56-A), 둘째, 안전보건 대표자 또는 안전보건 위원회(Q56-B), 셋째, 회사 내 안전 조직, 안전팀 또는 안전문제를 다룰 수 있는 창구(Q56-C), 네째, 직원이 회사에서 발생하는 일에 의견을 밝힐 수 있는 정기회의(Q56-D). 우리는 고위험 직업군 종사자를 고용형태에 따라 사업주, 자영업자, 무급가족종사자, 상용/임시/일용노동자로 구분하여 부문별, 성별 분포를 확인하고, 고위험 직업군 임금근로자를 대상으로 부문별, 성별, 고용형태에 따라 보호자원 보유 비율을 산출하였다.

**결과**

직업별 감염위험 중위값은 <표 1>에서 확인할 수 있다. 감염위험 중위값이 3점 이상인 고위험 직업군은 보건의료복지 7개 직업군, 그 외 부문 23개 직업군이다. 보건의료복지 부문에 속한 직업군들은 영양사를 제외하고 감염위험 중위값이 모두 5점 이상으로 높았다. 즉, 대부분의 보건의료복지 직업군은 거의 모든 근무시간에 고객, 승객, 학생, 환자처럼 직장동료가 아닌 사람들을 직접 상대하는 업무가 포함된다는 의미다. 그 외 부문의 경우, 종교관련 종사자, 교육전문가 및 관련직, 금융 및 보험사무직, 상담통계안내 및 기타 사무직, 이미용예식 서비스직, 운송 및 여가 서비스직, 조리 및 음식 서비스직, 영업직, 매장판매직, 방문노점 및 통신판매 관련직, 운송관련 단순노무직에서 감염위험 중위값이 5점 이상으로 높았다. 이처럼 고위험 직업군에는 의사, 간호사처럼 고위험 직업으로 사회적으로 인정받고 개인안전장비 등의 보호를 받는 직업 뿐만 아니라, 고위험 직업으로 인식되지 않는 요양/간병종사자, 서비스직 종사자, 교사 등이 포함되어 있었다.

대부분의 직업군에서 성별 분리가 뚜렷하게 관찰되었다<표 1>. 예컨대, 보건의료복지 부문에서 여성의 비율이 의료진료 전문가(의사)의 경우 25.1%로 낮은 반면, 간호사, 의료복지 관련 서비스 종사자(요양/간병종사자), 사회복지관련 종사자(사회복지사, 보육 교사 등), 보건의료관련 종사자(응급구조사, 간호조무사 등)에서는 각각 96.5%, 92.3%, 85.1%, 84.9%로 높았다. 그 외 부문에서도 여성 비율이 운전 및 운송 관련직, 운송 및 기계 관련 기능직, 영상 및 통신 장비 관련 기능직, 경찰소방 및 보안 관련 서비스직, 운송관련 단순 노무직(하역 및 적재 단순종사원, 음식배달원 등)에서 각각 2.1%, 6.3%, 4.0%, 10.9%, 12.7%로 매우 낮은 반면, 이미용·예식 서비스직, 가사음식 및 판매 관련 단순노무직, 상담통계안내 및 기타 사무직, 교육 전문가 및 관련직에서 각각 79.9%, 76.0%, 68.1%, 67.9%로 높았다. <그림 1>에서 직업군별 여성 종사자 비율과 감염위험 중위값이 양의 상관관계를 보인다 (R=0.4893, R2=0.2395, p<0.05). 여성의 비율이 높은 직업군은 대부분 고위험 직업군으로 분류되었다.

표 1. 직업군별 감염위험 중위값와 종사자 인구 규모 추정

| 표준직업분류 | 위험점수^1^ | 전체(명)^2^ | 여성(명)^2^ | 여성(%)^2^ |
| --- | --- | --- | --- | --- |
| 보건의료복지 (3자리 분류) |  |  |  |  |
| 의료진료 전문가 (의사) | 6 | 145,878 | 36,574 | 25.1 |
| 약사 및 한약사 | 5 | 35,541 | 21,232 | 59.7 |
| 간호사 | 5 | 227,168 | 219,301 | 96.5 |
| 치료사 및 의료기사 | 5 | 158,096 | 105,461 | 66.7 |
| 보건의료관련 종사자 (응급구조사, 간호조무사) | 5 | 186,996 | 158,775 | 84.9 |
| 사회복지관련 종사자 | 5 | 430,185 | 366,009 | 85.1 |
| 의료복지 관련 서비스 종사자 (요양/간병종사자) | 5 | 222,830 | 205,581 | 92.3 |
| 영양사 | 1 | 37,812 | 36,228 | 95.8 |
| 비 보건의료복지 (2자리 분류) |  |  |  |  |
| 종교관련 종사자 | 5 | 111,556 | 30,016 | 26.9 |
| 교육 전문가 및 관련직 | 5 | 1,235,726 | 839,663 | 67.9 |
| 금융 및 보험 사무직 | 5 | 354,937 | 169,706 | 47.8 |
| 상담통계안내 및 기타 사무직 | 5 | 313,483 | 213,613 | 68.1 |
| 이미용예식 서비스직 | 5 | 308,603 | 246,724 | 79.9 |
| 운송 및 여가 서비스직 | 5 | 249,609 | 118,839 | 47.6 |
| 조리 및 음식 서비스직 | 5 | 1,415,853 | 942,284 | 66.6 |
| 영업직 | 5 | 737,803 | 210,655 | 28.6 |
| 매장 판매직 | 5 | 1,576,184 | 865,985 | 54.9 |
| 방문노점 및 통신판매 관련직 | 5 | 384,429 | 221,404 | 57.6 |
| 운송관련 단순노무직 | 5 | 426,099 | 54,186 | 12.7 |
| 경영금융 전문가 및 관련직 | 4 | 473,382 | 150,553 | 31.8 |
| 식품가공관련 기능직 | 4 | 178,102 | 89,709 | 50.4 |
| 섬유 및 신발 관련 기계조작직 | 4 | 138,160 | 50,227 | 36.4 |
| 농림어업 및 기타 서비스 단순노무직 | 4 | 544,583 | 249,937 | 45.9 |
| 전문서비스 관리직 | 3 | 109,368 | 29,549 | 27.0 |
| 법률 및 행정 전문직 | 3 | 64,662 | 13,248 | 20.5 |
| 법률 및 감사 사무직 | 3 | 81,923 | 29,676 | 36.2 |
| 경찰소방 및 보안 관련 서비스직 | 3 | 245,764 | 26,676 | 10.9 |
| 운송 및 기계 관련 기능직 | 3 | 358,365 | 14,284 | 4.0 |
| 영상 및 통신 장비 관련 기능직 | 3 | 65,052 | 4,105 | 6.3 |
| 운전 및 운송 관련직 | 3 | 868,592 | 17,887 | 2.1 |
| 가사음식 및 판매 관련 단순노무직 | 3 | 497,271 | 377,984 | 76.0 |
| 판매 및 고객서비스 관리직 | 2 | 54,666 | 10,288 | 18.8 |
| 문화예술스포츠 전문가 및 관련직 | 2 | 547,027 | 250,232 | 45.7 |
| 목재가구악기 및 간판 관련 기능직 | 2 | 73,208 | 9,383 | 12.8 |
| 전기 및 전자 관련 기능직 | 2 | 279,374 | 18,455 | 6.6 |
| 행정 및 경영지원 관리직 | 1 | 74,630 | 12,263 | 16.4 |
| 건설전기 및 생산 관련 관리직 | 1 | 45,269 | 2,784 | 6.1 |
| 과학 전문가 및 관련직 | 1 | 99,892 | 36,726 | 36.8 |
| 정보통신 전문가 및 기술직 | 1 | 367,406 | 60,871 | 16.6 |
| 공학 전문가 및 기술직 | 1 | 846,303 | 100,155 | 11.8 |
| 경영 및 회계 관련 사무직 | 1 | 3,171,132 | 1,387,086 | 43.7 |
| 농축산 숙련직 | 1 | 1,155,422 | 511,413 | 44.3 |
| 어업 숙련직 | 1 | 58,959 | 15,810 | 26.8 |
| 섬유의복 및 가죽 관련 기능직 | 1 | 221,280 | 130,616 | 59.0 |
| 금속성형관련 기능직 | 1 | 218,049 | 12,228 | 5.6 |
| 건설 및 채굴 관련 기능직 | 1 | 595,404 | 37,916 | 6.4 |
| 기타 기능 관련직 | 1 | 148,804 | 25,515 | 17.1 |
| 식품가공관련 기계조작직 | 1 | 121,563 | 50,295 | 41.4 |
| 화학관련 기계조작직 | 1 | 239,152 | 61,025 | 25.5 |
| 금속 및 비금속 관련 기계조작직 | 1 | 254,918 | 32,329 | 12.7 |
| 기계제조 및 관련 기계조작직 | 1 | 542,978 | 99,427 | 18.3 |
| 전기 및 전자 관련 기계조작직 | 1 | 440,371 | 136,174 | 30.9 |
| 상하수도 및 재활용 처리관련 기계조작직 | 1 | 37,583 | 3,572 | 9.5 |
| 목재인쇄 및 기타 기계조작직 | 1 | 197,719 | 59,849 | 30.3 |
| 건설 및 광업 관련 단순노무직 | 1 | 339,473 | 24,671 | 7.3 |
| 제조관련 단순노무직 | 1 | 123,769 | 71,679 | 57.9 |
| 청소 및 경비 관련 단순노무직 | 1 | 615,971 | 275,305 | 44.7 |
| 임업 숙련직 | 0 | 5,351 | 717 | 13.4 |
| 자료: ^1^제 5차 근로환경조사(2017). 직업군별 감염위험 중위값 (가중치 적용). ^2^인구총조사 20% 표본조사(2015). | | | | |


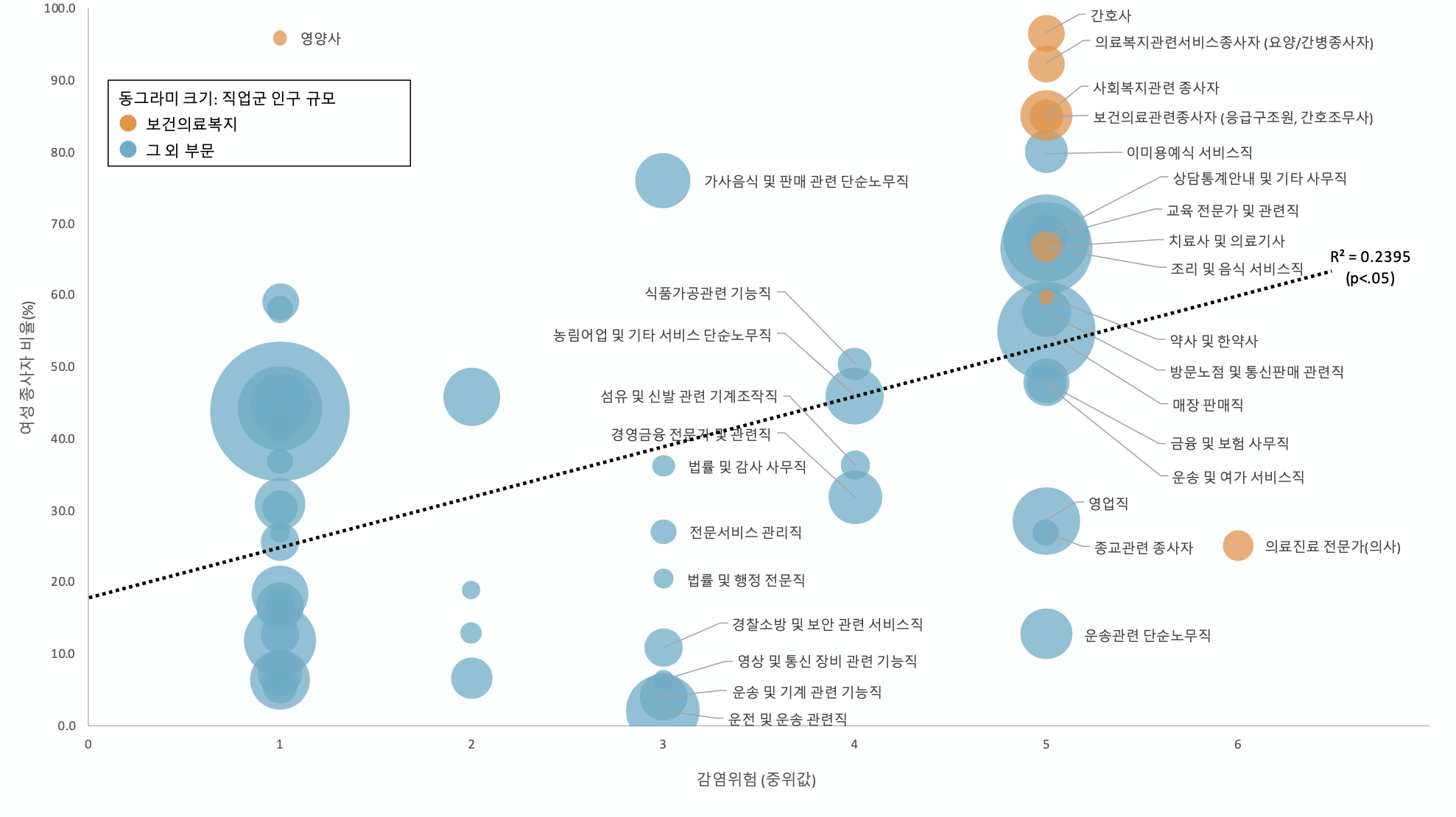


그림 1. 전체 직업별 감염위험 중위값과 여성 종사자 비율

<표 2>는 감염위험 중위값이 3점 이상인 고위험 직업군의 종사자 규모와 성별 구성, 월 평균 임금을 보여준다. 보건의료복지 부문에서 고위험 직업군 종사자는 총 140만 명이고, 79.1%가 여성이다. 그 외 부문의 경우, 고위험 직업군 종사자는 총 1,073만 명이고, 여성이 46.3%를 차지한다. <그림 2>에서 고위험 직업군의 여성 종사자 비율과 월 평균 임금은 음의 상관관계를 보인다 (R=0.4523, R2=0.2046, p<0.05). 감염 위험이 높은 직업군 안에서도 임금격차가 컸다. 여성 비율이 높은 직업군에서 월 평균 임금이 낮았다. 특히 요양/간병종사자는 92.3%가 여성인 전형적인 ‘여성 일자리’이고 감염 위험은 높지만, 월 평균 임금이 124만원으로 매우 낮았다. 가사음식 및 판매 관련 단순노무직(예: 가사 및 육아도우미) 또한 종사자의 76%가 여성이면서 월 평균 임금이139만원에 불과했다.

표 2. 감염 고위험 직업군 종사자 인구 규모 추정과 월 평균 소득

|  | 전체(명) ^1^ | 여성 종사자^1^ | | 월평균소득 (만원) ^2^ |
| --- | --- | --- | --- | --- |
| 감염 고위험 직업군^1^ |  | 명 | % |  |
| 보건의료복지 (3자리 분류) |  |  |  |  |
| 의료진료 전문가 (의사) | 145,878 | 36,574 | 25.1 | 581 |
| 약사 및 한약사 | 35,541 | 21,232 | 59.7 | 509 |
| 간호사 | 227,168 | 219,301 | 96.5 | 265 |
| 치료사 및 의료기사 | 158,096 | 105,461 | 66.7 | 286 |
| 보건의료관련 종사자 (응급구조사, 간호조무사) | 186,996 | 158,775 | 84.9 | 246 |
| 사회복지관련 종사자 | 430,185 | 366,009 | 85.1 | 218 |
| 의료복지 관련 서비스 종사자 (요양/간병종사자) | 222,830 | 205,581 | 92.3 | 124 |
| 고위험 직업군 종사자 | 1,406,694 | 1,112,933 | 79.1 |  |
| 비 보건의료복지 (2자리 분류) |  |  |  |  |
| 종교관련 종사자 | 111,556 | 30,016 | 26.9 | 202 |
| 교육 전문가 및 관련직 | 1,235,726 | 839,663 | 67.9 | 288 |
| 금융 및 보험 사무직 | 354,937 | 169,706 | 47.8 | 378 |
| 상담통계안내 및 기타 사무직 | 313,483 | 213,613 | 68.1 | 219 |
| 이미용예식 서비스직 | 308,603 | 246,724 | 79.9 | 250 |
| 운송 및 여가 서비스직 | 249,609 | 118,839 | 47.6 | 263 |
| 조리 및 음식 서비스직 | 1,415,853 | 942,284 | 66.6 | 258 |
| 영업직 | 737,803 | 210,655 | 28.6 | 343 |
| 매장 판매직 | 1,576,184 | 865,985 | 54.9 | 262 |
| 방문노점 및 통신판매 관련직 | 384,429 | 221,404 | 57.6 | 264 |
| 운송관련 단순노무직 | 426,099 | 54,186 | 12.7 | 260 |
| 경영금융 전문가 및 관련직 | 473,382 | 150,553 | 31.8 | 388 |
| 식품가공관련 기능직 | 178,102 | 89,709 | 50.4 | 279 |
| 섬유 및 신발 관련 기계조작직 | 138,160 | 50,227 | 36.4 | 276 |
| 농림어업 및 기타 서비스 단순노무직 | 544,583 | 249,937 | 45.9 | 149 |
| 전문서비스 관리직 | 109,368 | 29,549 | 27.0 | 540 |
| 법률 및 행정 전문직 | 64,662 | 13,248 | 20.5 | 674 |
| 법률 및 감사 사무직 | 81,923 | 29,676 | 36.2 | 374 |
| 경찰소방 및 보안 관련 서비스직 | 245,764 | 26,676 | 10.9 | 327 |
| 운송 및 기계 관련 기능직 | 358,365 | 14,284 | 4.0 | 347 |
| 영상 및 통신 장비 관련 기능직 | 65,052 | 4,105 | 6.3 | 336 |
| 운전 및 운송 관련직 | 868,592 | 17,887 | 2.1 | 311 |
| 가사음식 및 판매 관련 단순노무직 | 497,271 | 377,984 | 76.0 | 139 |
| 고위험 직업군 종사자 | 10,739,506 | 4,966,910 | 46.3 |  |
| 자료: ^1^인구총조사 20% 표본조사(2015). ^2^ 제 5차 근로환경조사(2017). 직업군별 월 평균소득의 평균값 (가중치 적용). | | | | |


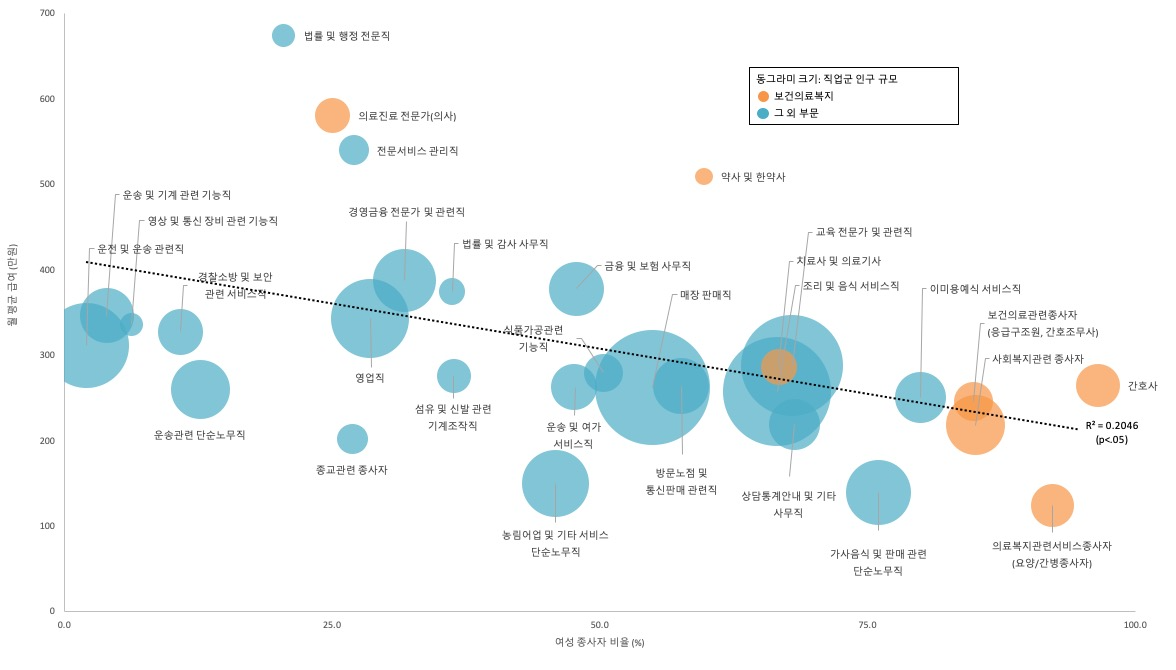


그림 2. 감염 고위험 직업의 여성 종사자 비율과 월 평균 임금

<표 3>은 고위험 직업군별로 ‘사람을 들어 올리거나 이동시키는 업무’가 근무시간 1/4 이상 포함된다고 응답한 종사자의 비율을 산출하여 고강도 위험에 노출되는 종사자 규모를 추정한 결과이다. 고위험 직업군 중 고강도 위험 노출 종사자는 보건의료복지 부문에서 54만 명(여성 46만 명, 84.7%), 그 외 부문에서 102만 명(여성 45만 명, 45.0%))으로 추정되었다. 보건의료복지 부문 고위험 직업군 중 여성이 지배적인 의료복지 관련 서비스 종사자(요양/간병종사자)와 간호사 직업에서 고강도 위험에 노출되는 종사자의 비율이 각각 68.6%, 44.2%로 높게 나타났다. 그 외 부문에서는 경찰소방 및 보안 관련 서비스직과 가사·음식 및 판매 관련 단순노무직(가사 및 육아 도우미, 음식관련 단순 종사원 등)에서 각각 25.1%, 19.1%가 근무 시간 1/4 이상 ‘사람을 들어 올리거나 이동시키는 업무’를 한다고 응답하였다.

표 3. 고위험 직업군의 고강도 위험 노출 규모 추정

| 감염 고위험 직업군^1^ | 고강도 위험 노출 남녀전체 (%)^1^ | 고강도 위험 노출 남녀전체 (명)^2^ | 고강도 위험 노출  여성 (명)^3^ |
| --- | --- | --- | --- |
| 보건의료복지 (3자리 분류) |  |  |  |
| 의료진료 전문가 (의사) | 17.7 | 25,844 | 6,480 |
| 약사 및 한약사 | 1.7 | 593 | 354 |
| 간호사 | 44.2 | 100,463 | 96,984 |
| 치료사 및 의료기사 | 31.3 | 49,437 | 32,978 |
| 보건의료관련 종사자 (응급구조사, 간호조무사) | 32.7 | 61,200 | 51,964 |
| 사회복지관련 종사자 | 36.6 | 157,324 | 133,854 |
| 의료복지 관련 서비스 종사자 (요양/간병종사자) | 68.6 | 152,944 | 141,105 |
| 고강도 위험 노출 종사자 |  | 547,806 | 463,719 (84.7%) |
| 비 보건의료복지 (2자리 분류) |  |  |  |
| 종교관련 종사자 | 2.9 | 3,258 | 877 |
| 교육 전문가 및 관련직 | 9.2 | 113,390 | 77,047 |
| 금융 및 보험 사무직 | 5.3 | 18,847 | 9,012 |
| 상담통계안내 및 기타 사무직 | 5.8 | 18,152 | 12,369 |
| 이미용예식 서비스직 | 12.4 | 38,222 | 30,558 |
| 운송 및 여가 서비스직 | 8.0 | 19,854 | 9,452 |
| 조리 및 음식 서비스직 | 8.9 | 125,369 | 83,436 |
| 영업직 | 4.5 | 33,154 | 9,466 |
| 매장 판매직 | 8.6 | 135,031 | 74,188 |
| 방문노점 및 통신판매 관련직 | 7.3 | 27,901 | 16,069 |
| 운송관련 단순노무직 | 12.6 | 53,556 | 6,811 |
| 경영금융 전문가 및 관련직 | 5.0 | 23,633 | 7,516 |
| 식품가공관련 기능직 | 12.1 | 21,585 | 10,872 |
| 섬유 및 신발 관련 기계조작직 | 12.9 | 17,873 | 6,497 |
| 농림어업 및 기타 서비스 단순노무직 | 7.3 | 39,591 | 18,170 |
| 전문서비스 관리직 | 8.2 | 8,969 | 2,423 |
| 법률 및 행정 전문직 | 2.9 | 1,848 | 379 |
| 법률 및 감사 사무직 | 4.3 | 3,485 | 1,262 |
| 경찰소방 및 보안 관련 서비스직 | 25.1 | 61,740 | 6,701 |
| 운송 및 기계 관련 기능직 | 13.2 | 47,471 | 1,892 |
| 영상 및 통신 장비 관련 기능직 | 9.9 | 6,423 | 405 |
| 운전 및 운송 관련직 | 12.3 | 106,435 | 2,192 |
| 가사음식 및 판매 관련 단순노무직 | 19.1 | 94,915 | 72,147 |
| 고강도 위험 노출 종사자 |  | 1,020,704 | 459,744 (45.0%) |
| 자료: ^1^제5차 근로환경조사(2017). ^2, 3^인구총조사 20% 표본조사(2015). ^2^직업별 근로인구 수(표2 참고)*고강도 위험 노출 비율. ^3^직업별 고강도 위험 노출 종사자 수(남녀전체)*여성 종사자 비율(표 2 참고) | | | |

<표 4>는 KWCS (2017)에서 감염 고위험 직업군의 성별, 고용형태별 분포를 보여준다. 모든 부문, 성별에서 상용노동자가 가장 빈번한 고용형태였다. 보건의료복지 부문은 다른 부문보다 상용노동자의 비율이 높았다(남녀 전체: 77.4% vs.50.9%). 보건의료복지와 그 외 부문 모두에서 남성은 여성보다 자영업자, 사업주 비중이 더 높았고, 여성은 무급가족종사자, 임시직, 일용직 비중이 더 높았다.

표 4. 고위험 직업군의 고용형태별 분포

|  | 전체 | | 남자 | | 여자 | |
| --- | --- | --- | --- | --- | --- | --- |
| 고용형태 | N | % | N | % | N | % |
| 보건의료복지 |  |  |  |  |  |  |
| 사업주 | 106,472 | 6.1 | 71,984 | 21.4 | 34,488 | 2.4 |
| 자영업자 | 41,606 | 2.4 | 28,353 | 8.4 | 13,253 | 0.9 |
| 무급가족노동자 | 4,104 | 0.2 | 0 | 0.0 | 4,104 | 0.3 |
| 상용노동자 | 1,356,084 | 77.4 | 216,140 | 64.2 | 1,139,944 | 80.5 |
| 임시노동자 | 202,193 | 11.5 | 19,390 | 5.8 | 182,803 | 12.9 |
| 일용노동자 | 42,401 | 2.4 | 621 | 0.2 | 41,780 | 2.9 |
| 전체 | 1,752,860 | 100 | 336,488 | 100 | 1,416,372 | 100 |
| 비 보건의료복지 |  |  |  |  |  |  |
| 사업주 | 1,033,972 | 8.2 | 704,234 | 10.8 | 329,739 | 5.4 |
| 자영업자 | 2,651,095 | 21.0 | 1,534,335 | 23.5 | 1,116,760 | 18.4 |
| 무급가족노동자 | 506,650 | 4.0 | 57,424 | 0.9 | 449,226 | 7.4 |
| 상용노동자 | 6,406,690 | 50.9 | 3,536,719 | 54.2 | 2,869,970 | 47.2 |
| 임시노동자 | 1,603,717 | 12.7 | 540,751 | 8.3 | 1,062,966 | 17.5 |
| 일용노동자 | 395,896 | 3.1 | 146,284 | 2.2 | 249,612 | 4.1 |
| 전체 | 12,598,019 | 100.0 | 6,519,747 | 100.0 | 6,078,272 | 100.0 |
| 자료: 제5차 근로환경조사(2017). 가중치 적용함. | | | | | | |

<표 5>는 고위험 직업군에 해당하는 임금노동자의 성별, 고용형태별 보호자원 보유율을 나타낸다. <표4>에서 응답자 수가 10건 미만인 보건의료복지 부문 남성 일용노동자(2명)는 분석에서 제외하였다. 모든 부문, 성별, 고용형태에서 보호자원이 있다고 응답한 노동자 비율이 낮았다. 보건의료복지 부문 여성 종사자는 노동조합 등 직원을 대표하는 유사위원회를 제외한 모든 보호자원이 있다고 응답한 노동자의 비율이 다른 부문 여성 종사자보다 높았다. 보건의료복지 부문 남성 종사자의 경우 고소득 전문직 의사가 대다수이기 때문에, 임시노동자의 보호자원 보유율이 상용노동자에 비해 높은 예외적인 결과가 나타났다. 사회적 지위가 높은 고소득 전문직은 일터에서 스스로의 건강과 안전을 보호할 수 있는 역량이 높기 때문에, 보호자원에 대한 접근이 고용형태의 제약에서 비교적 자유로울 수 있다. 하지만 여성 종사자의 경우, 고용 안정성이 높은 순서대로, 즉 상용, 임시, 일용노동자 순으로 보호자원 보유율이 높았다. 보건의료복지 부문 여성 종사자는 고소득 전문직보다는 저소득 전문직과 서비스직 비중이 높은 만큼, 보호자원은 고용 안정성과 직결된다.

보건의료복지 외 부문의 경우, 고용 안정성이 높은 상용노동자는 모든 보호자원, 즉 직원을 대표하는 유사위원회, 안전보건 대표자 또는 위원회, 회사 내 안전문제를 다룰 수 있는 창구, 직원이 회사에서 발생하는 일에 의견을 밝힐 수 있는 정기회의가 있다고 응답한 비율이 다른 고용형태에 비해 높다. 일용노동자는 남녀 모두에서 건강과 안전을 보호받을 수 있는 자원이 있다고 응답한 비율이 매우 낮았다. 동일한 고용형태라도 보호자원이 있다고 응답한 비율은 여성보다 남성에서 더 높았다.

표 5. 고위험 직업군 임금노동자의 고용형태별 보호자원 보유

|  |  | 보호자원 있음 (%) | | | |
| --- | --- | --- | --- | --- | --- |
| 성별 | 고용형태 | 직원을 대표하는 유사 위원회 | 안전보건 대표자/위원회 | 안전문제 해결 창구 | 직원 정기회의 |
| 보건의료복지 | | | | | |
| 전체 | 상용 | 11.6 | 18.0 | 22.3 | 27.9 |
|  | 임시 | 4.7 | 9.8 | 15.1 | 19.0 |
|  | 일용 | 1.5 | 3.3 | 3.4 | 2.5 |
| 남자 | 상용 | 12.5 | 18.5 | 24.5 | 31.4 |
|  | 임시 | 15.2 | 28.5 | 36.8 | 32.8 |
|  | 일용 | - | - | - | - |
| 여자 | 상용 | 11.5 | 17.9 | 21.9 | 27.3 |
|  | 임시 | 3.5 | 7.8 | 12.7 | 17.5 |
|  | 일용 | 1.6 | 3.3 | 3.4 | 2.5 |
| 비보건의료복지 |  |  |  |  |  |
| 전체 | 상용 | 16.7 | 14.7 | 21.1 | 27.6 |
|  | 임시 | 4.3 | 5.2 | 7.4 | 7.8 |
|  | 일용 | 2.0 | 3.7 | 5.2 | 5.7 |
| 남자 | 상용 | 20.6 | 18.1 | 25.4 | 31.2 |
|  | 임시 | 6.9 | 6.7 | 9.2 | 8.1 |
|  | 일용 | 3.7 | 6.4 | 9.1 | 9.4 |
| 여자 | 상용 | 12.0 | 10.6 | 15.7 | 23.3 |
|  | 임시 | 3.0 | 4.5 | 6.5 | 7.7 |
|  | 일용 | 1.0 | 2.2 | 3.0 | 3.6 |
| 자료: 제5차 근로환경조사(2017). 가중치 적용함. | | | | | |

**토론**

본 연구는 근로환경조사의 ‘환자 및 대중 직접 상대’ 변수를 감염위험 지표로 이용하여 코로나19와 같은 감염병에 취약한 고위험 직업군을 목록화하고 종사자 규모를 추정했다. 환자를 직접 상대하는 보건의료복지 부문의 7개 직업군과 그 외 부문 23개 직업군 종사자들이 근무 시간 절반 이상을 직장동료가 아닌 대중을 상대하는 업무를 수행하고 있었다. 30개 고위험 직업군 중 고강도 위험에 노출되는 종사자의 규모가 보건의료복지 부문 54만 명, 그 외 부문 102만 명으로 추정되었다. 이러한 결과는 고위험 직업군의 사업장에 대해 정부가 안전보건 관리·감독·규제 및 지원 체계를 수립하고, 방역대책의 중심에 노동자의 안전과 건강 보호를 두는 관점의 전환이 필요함을 시사한다. 우선 당장 역학조사 과정에서 직업관련 요소를 자세히 확인하고, 직업관련 감염에 대한 모니터링과 그에 걸맞는 대책을 마련해야 한다.

해외의 연구들에서도 유사한 감염 고위험 직업군이 확인되었다. Baker 등[5]은 ‘한 달에 한번 이상 감염병 혹은 질병에 노출’되는 정도를 지표로 이용하여 미국에서 직업별 감염위험을 산출하였는데, 본 연구와 마찬가지로 의사, 간호사, 보건의료 지원 종사자를 포함하는 보건의료 종사자가 고위험 직업으로 나타났고, 그 외 경찰소방 및 보안관련 서비스직, 돌봄 서비스직, 사회복지 관련 종사자가 포함되었다. 아시아 6개국을 대상으로 한 연구[13]에서 감염병 발생 초기 고위험 직업군은 서비스 및 판매 종사자, 운전원, 공사현장 인부, 종교관련 종사자로 나타났고, 이후 고위험 직업군에는 보건의료 종사자, 청소 및 가사 종사자, 경찰관, 종교관련 종사자가 포함되었다.

본 연구는 감염 위험을 나타내는 여러 직업 특성 중 ‘환자 및 대중 노출’에 주목하였기 때문에, 모든 감염 고위험 직업군을 포괄하지 못하는 제한점이 있다. 코로나19는 환자 및 대중 노출뿐 아니라 밀집도가 높은 환경에서 빠르게 전파될 수 있다. 예컨대 집단 감염이 발생한 콜센터, 물류센터는 환자나 대중과의 접촉보다는 밀집도 높은 노동환경이 전파의 주된 경로로 작동하였다. 이는 한국만의 현상이 아니다. 필리핀의 콜센터[14], 미국[15], 독일[16], 아일랜드[17], 캐나다[18] 등의 육류가공공장 집단감염 사례는 환자나 대중과의 접촉만이 아니라 사업장 내의 물리적 밀집도, 환기, 위생설비 등의 중요성을 보여준다. 하지만 한국 근로환경조사에서 이러한 요소들을 확인할 수 있는 변수가 없었다. 감염 고위험 사업장을 선제적으로 발굴하고 예방조치를 취하기 위해서는 사업장 내 밀집도와 환기요소를 평가할 수 있는 정보가 추가적으로 필요하다. 코로나19와 미래에 발생할 새로운 감염병에 대비해서 사업장 내 밀집도를 포함한 노동환경에 대한 보다 세밀한 조사가 필요하다.

또한 아직 고위험 직업군으로 평가되지 않았지만 감염위험이 높게 나타날 가능성도 주목해야 한다. 예컨대, 미국 뉴헤이븐 하수처리장 분변 샘플에서 SARS-CoV-2 RNA가 확인되었고 지역사회 코로나 입원율, 검사율과 높은 상관관계를 보였다[19]. 이러한 결과는 하수처리장 모니터링을 통해 코로나19의 지역사회 전파를 예측할 수 있다는 함의와 더불어, 하수처리장 노동자들의 감염 위험을 시사하기도 한다. 실제 KWCS(2017)에서 직업군별 ‘폐기물, 체액, 실험 물질같이 감염을 일으키는 물질을 취급하거나 직접적인 접촉에 노출되는 정도’에서 근무시간 1/4 이상 노출되는 직업군은 ‘상하수도 및 재활용 처리관련 기계조작직’이 유일했다. 이처럼 대중이나 환자 같은 직접적 대인접촉이 없더라도 감염위험에 노출될 수 있는 직종에 대한 고려도 필요하다.

고위험 직업군의 성별 구성과 일자리 특성, 보호자원의 분포를 이해하는 것도 중요하다. 여성이 과반수 이상을 차지하는 전형적 여성 일자리의 대부분이 고위험 직업군이었다. 그리고, 고위험 직업군 중 중위소득(2020년 1인 기준중위소득 175만 원)을 밑도는 저임금 일자리인 요양/간병종사자, 가사음식 및 판매관련 단순노무직(예: 가사 및 육아도우미)도 대부분이 여성이 수행한다. 이러한 저임금, 고위험 직업군의 노동의 가치는 포스트 코로나 시대 새롭게 평가되어야 하며, 이들의 보호에 대한 고려가 절실하다.

한편 본 연구에서 노동자를 대표하고 노동자의 권익을 위해 활동하는 조직이 고위험 직업군 노동자에게 불평등하게 분배되어 있음이 드러났다. 고용이 불안정한 임시직, 일용직 노동자는 보호자원이 없는 비율이 높았다. 현재 산업안전보건법은 상시근로자 100인 이상 사업장(유해위험업종은 업종에 따라 50명 이상, 100명 이상)에 대해서만 안전보건관리책임자를 두고, 산업안전보건위원회를 설치하도록 한다. 고위험 직업군의 불안정 노동자들은 노동안전보건 규제의 사각지대에서 보호받기 힘들다. 불안정고용, 비정규직에 의존하는 한국의 고용구조는 감염병과 같은 위험에 취약할 수 밖에 없고, 이는 일터에서 취약노동자의 건강피해로 끝나지 않고 지역사회 전파로 이어져 감염병 통제를 어렵게 한다. 노동자가 불리한 대우에 대한 걱정없이 안전 문제를 제기하고 보호받을 수 있는 창구가 보장된다면 일터를 안전하게 만들고 지역사회 전파위험을 줄일 수 있을 것이다.

코로나19를 계기로 ‘위험한 일’에 대한 패러다임이 변화하고 있다. KWCS(2017)에서 직업군별로 자신의 일이 ‘건강을 해치거나 안전상 위험한 일’이라고 응답한 사람의 비율을 확인한 결과(Supplementary material 1), 전통적으로 사고성 산업재해가 빈번한 제조업, 건설업 등이 위험한 일로 인식되고 있었다. 금속성형관련 기능직(용접원 등), 건설 및 광업 관련 단순노무직, 어업 숙련직 등에서 절반에 가까운 노동자가 자신의 일을 ‘위험한 일’이라고 응답했다. 본 연구에서 감염 고위험 직업군으로 확인된 대부분의 직업군에서 자신의 일이 ‘건강을 해치거나 안전상 위험한 일’이라고 응답한 비율은 10% 미만이었다. 분명한 것은 코로나19가 종식된다고 해서 고위험 직업군이 저위험 직업군으로 바뀌는 것이 아니라는 점이다. 코로나19는 ‘환자 접촉’, ‘대중 접촉’, ‘밀집도 높은 노동환경’ 등 지금까지 사회적으로 인정받지 못한 ‘위험’을 드러내고 사회적 보호의 필요성을 대두시킨 것이다.

**Acknowledgements**

This work was made possible due to the support of the Vanier Canada Graduate Scholarships received by Juyeon Lee.

**Conflict of Interest**

The authors have no conflicts of interest to declare for this study.

**References**

1. Korea Central Disaster Management Headquarters. Coronavirus Disease-19, Republic of Korea [cited 2020 June 2]. Available from: <http://ncov.mohw.go.kr/baroView2.do?brdId=4&brdGubun=42>.
2. ILO. ILO Monitor: COVID-19 and the world of work. [cited 2020 June 2]. Available from: <https://www.ilo.org/wcmsp5/groups/public/@dgreports/@dcomm/documents/briefingnote/wcms_745963.pdf>.
3. Ki M. 2015 MERS outbreak in Korea: Hospital-to-hospital transmission. Epidemiology and health 2015;37:e2015033-e2015033.
4. PHI. Human rights-based approach to risk management: People’s perspective on 2015 MERS outbreak in Korea (Korean, author’s translation) [cited 2020 June 2]. Available from: <http://health.re.kr/?p=2751>.
5. Baker MG, Peckham TK, Seixas NS. Estimating the burden of United States workers exposed to infection or disease: A key factor in containing risk of COVID-19 infection. PLOS ONE 2020;15:e0232452.
6. Gamio L. The workers who face the greatest Coronavirus risk [cited 2020 June 2]. Available from: <https://www.nytimes.com/interactive/2020/03/15/business/economy/coronavirus-worker-risk.html>.
7. Lu M. The front line: Visualizing the occupations with the highest COVID-19 risk [cited 2020 June 2]. Available from: <https://www.visualcapitalist.com/the-front-line-visualizing-the-occupations-with-the-highest-covid-19-risk/>.
8. Macdonald D. Between a rock and a hard place: Which workers are most vulnerable when their workplaces re-open amid COVID-19? [cited 2020 June 2]. Available from: <http://behindthenumbers.ca/2020/05/15/between-a-rock-and-a-hard-place-which-workers-are-most-vulnerable-when-their-workplaces-re-open-amid-covid-19/>.
9. Frogner BK. How many health care workers are at risk of being sacrificed to COVID-19 in the US? [cited 2020 June 2]. Available from: <http://depts.washington.edu/fammed/chws/how-many-health-care-workers-are-at-risk-of-being-sacrificed-to-covid-19-in-the-us/>.
10. Watterson A. COVID-19 in the UK and Occupational Health and Safety: Predictable not Inevitable Failures by Government, and Trade Union and Nongovernmental Organization Responses. NEW SOLUTIONS: A Journal of Environmental and Occupational Health Policy 2020;0:1048291120929763.
11. Lay AM, Saunders R, Lifshen M, Breslin FC, LaMontagne AD, Tompa E, et al. The relationship between occupational health and safety vulnerability and workplace injury. Safety Science 2017;94:85-93.
12. Lay AM, Saunders R, Lifshen M, Breslin C, LaMontagne A, Tompa E, et al. Individual, occupational, and workplace correlates of occupational health and safety vulnerability in a sample of Canadian workers. Am J Ind Med 2016;59:119-128.
13. Lan F-Y, Wei C-F, Hsu Y-T, Christiani DC, Kales SN. Work-related COVID-19 transmission in six Asian countries/areas: A follow-up study. PLOS ONE 2020;15:e0233588.
14. Galant M. Philippine call center workers are in danger. It’s our problem, too. [cited 2020 June 2]. Available from: <https://inthesetimes.com/working/entry/22547/philippine_call_center_workers_covid_cwa_unions_corporate_globalization>.
15. Coleman J. Meatpacking worker told not to wear face mask on job died of coronavirus: report. Available from: <https://thehill.com/policy/finance/496595-meatpacking-worker-told-not-to-wear-face-mask-on-job-died-of-coronavirus?fbclid=IwAR0NFGOQmVkHcLFJ6szp8DQV3VoKUeb2rVe5ZPjuvu1WIMus_sAioNOZK3Q>.
16. Coronavirus outbreak closes German meat-packing plant [cited 2020 June 2]. Available from: <https://www.dw.com/en/coronavirus-outbreak-closes-german-meat-packing-plant/a-53374478>.
17. McSweeney E. Covid-19 outbreaks at Irish meat plants raise fears over worker safety [cited 2020 June 2]. Available from: <https://www.theguardian.com/environment/2020/may/01/covid-19-outbreaks-at-irish-meat-plants-raise-fears-over-worker-safety>.
18. Harris C. Cargill meat-processing plant south of Montreal says 64 workers infected with COVID-19 [cited 2020 June 2]. Available from: <https://www.cbc.ca/news/canada/montreal/cargill-chambly-covid-19-shut-down-1.5563539>.
19. Peccia J, Zulli A, Brackney DE, Grubaugh ND, Kaplan EH, Casanovas-Massana A, et al. SARS-CoV-2 RNA concentrations in primary municipal sewage sludge as a leading indicator of COVID-19 outbreak dynamics. medRxiv 2020:2020.2005.2019.20105999.

Supplementary material 1. 자신의 일이 ‘건강을 해치거나 안전상 위험한 일’이라고 응답한 사람의 직업별 비율

| 표준직업분류 | % | 표준직업분류 | % |
| --- | --- | --- | --- |
| 금속성형관련 기능직 | 49.77 | 조리 및 음식 서비스직 | 9.45 |
| 건설 및 광업 관련 단순노무직 | 49.74 | 의료복지 관련 서비스 종사자 | 9.45 |
| 어업 숙련직 | 45.34 | 간호사 | 8.70 |
| 건설 및 채굴 관련 기능직 | 37.21 | 가사음식 및 판매 관련 단순노무직 | 8.26 |
| 금속 및 비금속 관련 기계조작직 | 35.81 | 의료진료 전문가 | 8.09 |
| 운전 및 운송 관련직 | 35.79 | 농림어업 및 기타 서비스 단순노무직 | 7.82 |
| 운송관련 단순노무직 | 33.69 | 이미용예식 서비스직 | 7.51 |
| 임업 숙련직 | 32.45 | 운송 및 여가 서비스직 | 7.41 |
| 경찰소방 및 보안 관련 서비스직 | 31.33 | 법률 및 행정 전문직 | 7.22 |
| 상하수도 및 재활용 처리관련 기계조작직 | 30.79 | 보건의료관련 종사자 | 6.91 |
| 기타 기능 관련직 | 29.01 | 제조관련 단순노무직 | 5.31 |
| 전기 및 전자 관련 기능직 | 26.40 | 문화예술스포츠 전문가 및 관련직 | 4.72 |
| 운송 및 기계 관련 기능직 | 23.55 | 매장 판매직 | 4.48 |
| 목재가구악기 및 간판 관련 기능직 | 22.42 | 영업직 | 4.16 |
| 기계제조 및 관련 기계조작직 | 21.90 | 정보통신 전문가 및 기술직 | 3.99 |
| 영상 및 통신 장비 관련 기능직 | 21.12 | 종교관련 종사자 | 3.68 |
| 화학관련 기계조작직 | 19.80 | 사회복지관련 종사자 | 2.98 |
| 목재인쇄 및 기타 기계조작직 | 18.66 | 상담통계안내 및 기타 사무직 | 2.86 |
| 전기 및 전자 관련 기계조작직 | 15.31 | 경영 및 회계 관련 사무직 | 2.60 |
| 과학 전문가 및 관련직 | 14.71 | 영양사 | 2.58 |
| 섬유 및 신발 관련 기계조작직 | 13.66 | 방문노점 및 통신판매 관련직 | 2.52 |
| 건설전기 및 생산 관련 관리직 | 13.10 | 경영금융 전문가 및 관련직 | 2.48 |
| 농축산 숙련직 | 13.03 | 약사 및 한약사 | 2.35 |
| 공학 전문가 및 기술직 | 11.99 | 금융 및 보험 사무직 | 2.25 |
| 섬유의복 및 가죽 관련 기능직 | 11.55 | 교육 전문가 및 관련직 | 2.00 |
| 식품가공관련 기능직 | 11.50 | 전문서비스 관리직 | 1.85 |
| 식품가공관련 기계조작직 | 11.19 | 법률 및 감사 사무직 | 1.60 |
| 치료사 및 의료기사 | 11.12 | 판매 및 고객서비스 관리직 | 0 |
| 청소 및 경비 관련 단순노무직 | 10.77 |  |  |
| 행정 및 경영지원 관리직 | 10.10 |  |  |
| 자료: 제 5차 근로환경조사(2017). 가중치 적용. | | | |

1. Note

   한국 근로환경조사(KWCS, Korean Working Conditions Survey)는 유럽근로 환경조사(EWCS)와 영국 노동력조사(LFS, Labour Force Survey)를 벤치마킹하여 직종, 업종, 고용형태, 근로환경 위험요인 노출 등 업무환경을 전반적으로 파악하기 위한 조사이다. 표본은 1단계에서 조사구를 추출하고 2단계에서 가구 및 가구원을 추출하는 2단계 확률비례 층화집락추출(Secondary probability proportion stratified cluster sample survey)에 따라 설계되었다. [↑](#endnote-ref-1)
2. 근로환경조사는 표본수가 5만 명으로 매우 적기 때문에 직업군별 모수를 추정하는 데 상당한 불확실성이 있다. 따라서, 본 연구의 목적인 고위험 직업군의 ‘실제 규모’를 추정하기 위해서 전국 가구의 20% 가구, 대략 1,000만 명을 표본으로 선정하여 조사하는 2015년 인구총조사 20% 표본조사가 더 정확하다고 판단하였다. 근로환경조사에서 표본수가 비교적 많은 직업군의 경우 인구총조사 표본조사에서 추정한 직업군 규모보다 크게 나타난 반면, 표본수가 적은 직업군의 경우 인구총조사 표본조사에서 추정한 직업군 규모보다 적게 나타났다. [↑](#endnote-ref-2)
